# Frailty and prediction of recurrent falls over 10 years in a community cohort of 75-year-old women

**DOI:** 10.1007/s40520-019-01467-1

**Published:** 2020-01-14

**Authors:** Patrik S. Bartosch, Jimmie Kristensson, Fiona E. McGuigan, Kristina E. Akesson

**Affiliations:** 1 Clinical and Molecular Osteoporosis Research Unit, Department of Clinical Sciences Malmö, Lund University, Lund, Sweden; 2 Department of Orthopaedics, Skåne University Hospital, IM Nilssonsgata 22, 205 02 Malmö, Sweden; 3 Department of Health Science, Lund University, Lund, Sweden

**Keywords:** Frailty, Falls, Women, Community-dwelling

## Abstract

**Background:**

Frailty captures the age-related declines in health leading to increased vulnerability, including falls which are commonplace in older women. The relationship between frailty and falls is complex, with one leading to the other in a vicious cycle.

**Aims:**

This study addresses the gap in understanding how patterns of frailty and falls propensity interact, particularly in those who have *not yet* entered the falls-frailty cycle.

**Methods:**

The Osteoporosis Risk Assessment cohort consists of 1044 community-dwelling women aged 75, with 10 years of follow-up. Investigations were performed and a frailty index constructed at baseline, 5 and 10 years. Falls were self-reported for each previous 12 months. Analysis was two-directional, firstly based on frailty status and second, based on falls status. Recurrent falls was the primary outcome.

**Results:**

Baseline frailty was a significant predictor of recurrent falls after 5 and 10 years [(OR 2.55 (1.62–3.99); 3.04 (1.63–5.67)]. Among women who had *no history* of falls at age 75, frailty was a stronger predictor of falls at 5 years [OR 3.06 (1.59–5.89)] than among women who had previously fallen.

**Discussion:**

Frailty is significantly associated with recurrent falls and most pronounced in those who are frail but have not yet fallen.

**Conclusions:**

This suggests that frailty should be an integral part of falls-risk assessment to improve identification of those at risk of becoming fallers.

**Electronic supplementary material:**

The online version of this article (10.1007/s40520-019-01467-1) contains supplementary material, which is available to authorized users.

## Introduction

Frailty, the age-related decline in reserve capacity and resilience, is associated with a multitude of adverse outcomes [1]. Deficits in musculoskeletal health contributes to frailty with gait problems, weakness, reduced reaction time and balance, factors also leading to falls risk [2, 3]. The consequences of falls leads to extensive costs from injuries and fractures, disability and nursing home placement [4]. Given the demographic shift towards an older population and anticipated high care burden, frailty is a research priority.

The relationship between frailty and falls is demonstrated by observations that in community-dwelling populations aged 65 and over, every third person experiences at least one fall annually; fifteen percent leading to significant injury [5–7]. In those over 80, the proportion increases to every second person. Causes of falling are complex and the combination of general health status, environmental circumstances and chance makes prediction difficult [8]. Fall specific scales have been developed; however, their predictive ability is limited and the clinical utilization neither consistent or widespread [9, 10].

Assessment of frailty may capture the multi-factorial aspects of falls propensity. Since an important objective is identifying individuals *before* they become frail, this opens the possibility to capture an elevated falls risk before it manifests clinically.

Previous falls are important predictors of future falls [3]; however, the correlation between *frailty* and falls is also high [11, 12], although difficult to untangle as they are reciprocal. This ‘vicious cycle’ of functional decline with frailty leading to falls, greater frailty and more falls, makes it imperative to understand *if* and *how* frailty affects those who have *not yet* suffered a fall compared to those already in the falls-frailty cycle. Regardless if frailty precedes falls or vice versa, interventions, whether physical or nutritional, are more likely to be effective before a point-of-no-return is reached [13].

A clear picture of the frailty–falls relationship is difficult to obtain, not least due to differences in study design and frailty and falls measures. Most studies utilize a categorical frailty definition [1]; however, this could hamper assessment of a gradually higher frailty and its association to falls. Therefore, the ambition of this study was to create a continuous deficit accumulation frailty index [14] with which to investigate this relationship.

An additional gap-in-knowledge is the time frame of prediction; 3 months to 5 years is well studied [6, 15], while little is known in a longer perspective. This is an important aspect since maintaining a good quality of life during aging is related to *not* entering the frailty–falls cycle. In a previous study we followed the progression of frailty over 10 years in the Osteoporosis Risk Assessment (OPRA) cohort and its association with osteoporosis [16].

In the present study the overall aim is to understand frailty and its relationship to fall propensity in short and longer perspectives. Seventy-five is a pivotal age at which most are still physically active and relatively healthy; therefore, the consequences of a fall, especially if a fracture results, often marks the beginning of a more dependent state.

Our specific aims were to (1) describe the proportion who are frail at age 75, 80 and 85 and the number reporting recent falls, (2) determine the association between frailty and risk of recurrent falls, (3) determine if a gradual increase in frailty is associated with the number of future falls and (4) explore the relationship of frailty to future falls in women with or without previous falls.

## Materials and methods

### Subjects

The OPRA cohort consists of 75-year-old community-dwelling women (75.2 ± 0.2) as described in detail [17]. *N* = 1044 attended baseline. Detailed follow-up investigations including questionnaires, physical and falls assessment were performed at 5 years (*n* = 715, age 80.2 ± 0.2) and 10 years (*n* = 382, age 85 ± 0.1) [18, 19].

Participants provided written informed consent. The regional ethical review board in Lund approved the study (Dnr:2014804), which was performed according to the Helsinki Declaration principles.

### Frailty index

We constructed a frailty index [16, 17] adhering to the principles of Searle et al. [20]. Briefly, the index includes thirteen variables covering a number of physiological domains (daily physical activity, time spent out-doors, walking speed, number of steps taken, balance, muscle strength, diabetes, cancer, diseases affecting balance, self-reported fall risk, polypharmacy, CRP and creatinine). The index represents the number of ‘deficits in health’ (scored 0.0–1.0); a higher score indicating higher frailty.

Since some variables in the index are dichotomized, loss-of-discrimination is possible (due to many individuals having identical values), therefore, as a refinement we reclassified each applicable variable as continuous between 0.0 and 1.0, i.e., providing a range. For example, “number of steps taken to walk 30 m”. Dichotomized, cut-points were < 54 steps = 0 or ≥ 54 steps = 1. Reclassifying this as a continuous variable, fewer steps indicates a longer stride, hence a healthier state and a score closer to zero. To implement this we examined the range of values across the entire cohort (in this case 21–160) and, after excluding extreme outliers, the highest (*V*_max_) and lowest (*V*_min_) values were set to 1 and 0, respectively. The original values (*V*_*x*_) were then reclassified using $\frac{{\left( {V_{x} - V_{\text{min} } } \right)}}{{\left( {V_{\text{max} } - V_{\text{min} } } \right)}}$.

To test how this 13-variable index related to a more typical index comprising dichotomized variables, we compared it to a 40-variable frailty index that had been created for the two follow-up visits [16]. The refined 13-variable index was highly correlated to the full 40-variable index (*r* = 0.80) and distributions were comparable (5 years: 0.24 vs. 0.23, _median_0.21 vs. 0.21; 10 years: 0.27 vs. 0.29, _median_0.26 vs. 0.27).

We used an empirical cutoff ≥ 0.25 to define frail individuals. This is suggested by others [21, 22] and supported through calculations in our cohort; plotting differences in 10-year mortality using 0.02 increments, the beginning of a steeper slope in the curve occurs at approximately 0.25.

### Falls

At baseline, 5-year and 10-year follow-up visits participants provided information on whether they had fallen in the previous 12 months and if they had fallen, how many times they fell during that period. In the analysis we define falls variously: at least one fall, recurrent falls (i.e., 2 or more falls) during the previous 12 months, the rationale being that multiple falls are more likely due to a frail disposition, mirroring a “falling-phenotype”. We also define women as ‘fallers’ and ‘non-fallers’ and we use ‘number of falls’. Only participants with valid data on falls were included (75 y *n* = 914; 80 y *n* = 711; 85 y *n* = 382).

### Statistical analyses

Descriptives are reported as mean (SD), median (IQR) and frequency (%). Comparisons of demographic characteristics, overall and between frail/non-frail categories, used Student’s *T* test and Chi square. The frailty index showed a typical skewed distribution at all timepoints [14] (tending towards normality at 10-year follow-up); non-parametric analyses were performed when appropriate.

Frailty was analysed primarily as ‘non-frail’ (≤ 0.25); ‘frail’ (> 0.25). To facilitate comparison with other studies, frailty was also used as a continuous variable in 0.01 increments. To explore a gradual increase in frailty, frailty quintiles were created.

To explore the relationship between frailty, at least one fall and recurrent falls, odds ratios (OR) with 95% confidence intervals were calculated using binary logistic regression, with adjustment for 25(OH)D, BMI, smoking and previous fractures (between 50 and 75 y) also performed.

To explore the relationship between frailty and falls status, we defined ‘fallers’ as those reporting at least one fall during the 12 months prior to baseline. We combined this with frailty status to give four groups (faller/frail; faller/non-frail; non-faller/frail; non-faller/non-frail); compared using cross tabulation, Chi square and regression analysis.

To explore the association between frailty at baseline (75 y) and number of future falls at 5-year follow-up, four groups were used (no falls, 1 fall, 2 falls, 3 or more falls). The same groups were used for comparison of frailty at age 80 and number of future falls at the next 5-year follow-up (85 y). Frailty was also binned into equal-sized quintiles and compared using cross tabulation. Only individuals who participated *and* had fall data at follow-up were included.

Analyses were performed using SPSS v25 and JMP (SAS Institute, USA). *P* < 0.05 was considered nominally significant.

## Results

Table 1 presents key clinical characteristics of the OPRA cohort at ages 75, 80 and 85. Table 2 presents key baseline characteristics of frail and non-frail women. The prevalence of frailty increased from 23.5% at baseline to 39.3% and 56.8% at 5 and 10-year follow-up, respectively. This is reflected in the median frailty score increasing with age; baseline 0.16 (_mean_0.19) and 0.21 (_mean_0.24) and 0.27 (_mean_0.29) at the 5- and 10-year follow-up.

**Table 1:** Key clinical characteristics of the OPRA cohort at age 75, 80 and 85

All variables at 75 y	Age 75 (Baseline) *n* = 1044		Age 80 (5 years) *n* = 715		Age 85 (10 years) *n* = 382	
	Mean	SD	Mean	SD	Mean	SD
Age (y)	75.2	(0.2)	80.2	(0.2)	85.2	(0.1)
Height (cm)	160.5	(5.7)	159.2	(5.8)	158.3	(5.8)
Weight (kg)	67.8	(11.7)	66.0	(11.6)	63.95	(10.9)
BMI (kg/m^2^)	26.3	(4.2)	26.1	(4.2)	25.5	(4.0)
S-25(OH)D (nmol/L)	62	(19)	78	(30)	79	(26)
Femoral Neck (T-score)	− 1.8	(1.1)	− 2.2	(1.1)	− 2.4	(1.1)

	Median	IQR	Median	IQR	Median	IQR
Frailty index (FI)	0.16	(0.14)	0.21	(0.17)	0.27	(0.20)

**Table 2:** Baseline characteristics of frail and non-frail women

All variables at 75 y	Non-frail (< 0.25)		Frail (≥ 0.25)		All Women	
	*n* = 799		*n* = 245		*n* = 1044	
	Median	IQR	Median	IQR	Median	IQR
Frailty index (FI)	0.14	(0.09)	0.32	(0.49)	0.16	(0.73)

	Mean	SD	Mean	SD	Mean	SD
BMI (Kg/m^2^)	26.0	(3.88)	27.0	(5.05)	26.3	(4.19)
S-25(OH)D (nmol/L)	63.1	(18.9)	57.7	(20.4)	61.8	(19.4)

	No	(%)	No	(%)	No	(%)
*Falls in previous 12* *months (n = 914)*						
1 fall	84	(12.2)	42	(19.0)	126	(13.8)
2 or more falls	62	(8.9)	72	(32.6)	134	(14.7)
No falls	547	(78.9)	107	(48.4)	654	(62.6)
*Prior fractures*						
Any (50–75 y)	278	(35.1)	105	(43.9)	383	(37.1)
Major osteoporotic (50–75 y)	187	(23.6)	53	(22.2)	240	(23.3)
*Education*						
Lower education	587	(73.6)	185	(76.4)	772	(74.2)
Higher education	211	(26.4)	58	(23.9)	269	(25.8)
*Smoking*						
Non-smoker	535	(67.6)	144	(59.8)	679	(65.7)
Previous	150	(18.9)	59	(24.5)	209	(20.2)
Current	107	(13.5)	38	(15.8)	145	(14.0)
*Alcohol*						
Abstainer	141	(17.8)	61	(25.6)	202	(19.6)
A few times a month	489	(61.2)	140	(58.8)	629	(60.9)
Weekly	149	(18.8)	31	(13.0	180	(17.4)
Almost daily	15	(1.9)	6	(2.5)	21	(2.0)

**Fig. 1:**
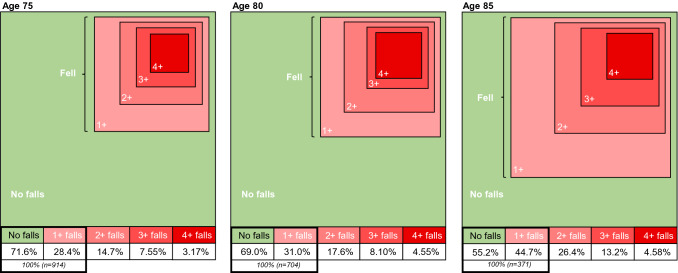
Proportion of women attending each visit who reported none, one or multiple falls in the previous 12 months. This figure shows how, with advancing age the proportion of women falling increases. At each visit (ages 75, 80 and 85) the proportion of women reporting haven fallen once or more in the previous 12 months increases from 28.4 to 31% to 44.7%. The green area represents non-fallers and shrinks as the proportion of women reporting falls increases. The fallers are represented with deepening shades of red to illustrate the multiple fallers; these increase over time as the proportion falling increases. Missing falls data: 75 y (*n* = 130); 80 y (*n* = 82)

The overall incidence of women reporting falls at each visit is illustrated in Fig. 1. At baseline, the proportion reporting at least one fall was 28.4% (*n* = 260), increasing to 31.0% (*n* = 218) and 44.7% (*n* = 166) at subsequent visits. A similar pattern is seen for recurrent falls; incidence almost doubles from age 75–85 (14.7%; 17.6%; 26.4%). Online_Resource _Figure 1 shows frailty score in relation to fall status at each visit.

**Fig. 2:**
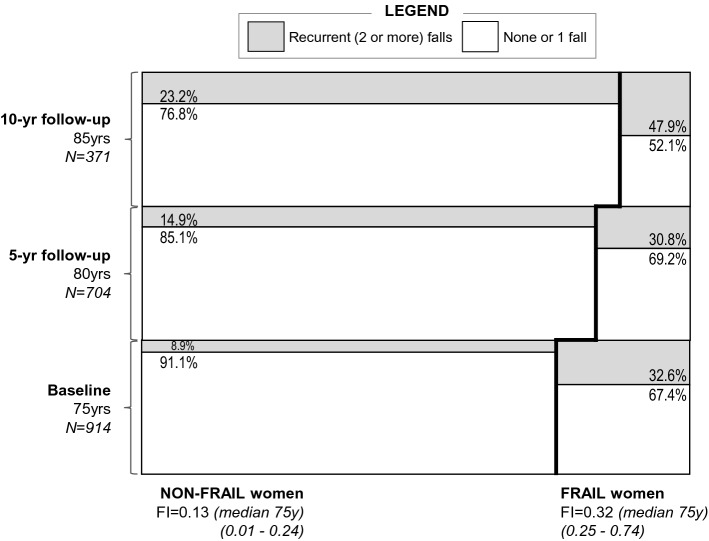
Proportion of non-frail and frail women women reporting recurrent falls at all visits based on frailty status at age 75. Women are defined as frail (≥ 0.25) or non-frail (< 0.25) at baseline and we show the proportion at each visit who reported recurrent falls in the previous 12 months. Among FRAIL women, proportionally more reported recurrent falls, compared to non-frail (32.6 vs. 8.9 at 75 y; 30.8 vs. 14.9 at 80 y; 47.9 vs. 23.2 at 85 y). Width of the frail segments narrows with successive visits, reflecting the proportionally higher loss-to-death and non-attendance in the most frail

Based on frailty status at 75, Fig. 2 illustrates the proportion of women who *did* or *did not* report recurrent falls in the previous 12-month period at 75, 80 and 85. At age 75, recurrent falls were almost four times more common among frail compared to non-frail women (32.6% vs. 8.9%; *p* < 0.001). Frail women continued to report recurrent falls across follow-up (5 y 30.8% vs. 14.9%; 10 y 47.9% vs. 23.2%, both *p* ≤ 0.001).

Baseline frailty was a significant predictor of recurrent falls. Calculating falls odds risk in relation to frailty status showed that being frail at age 75 was associated with increased falls risk up to 5 and 10 years; recurrent falls were 2.5–3 times more likely in frail vs. non-frail women [OR 2.55 (CI 1.62–3.99); 3.04 (1.63–5.67)] (Online_Resource_Table 1). Similar results were also observed assessing the relationship between frailty status at age 80 and fall risk after 5 years (85 years), with a two times higher OR compared to non-frail women.

At age 75 an increment of 0.01 in the index significantly increased the odds for at least one fall (1.04, 1.03–1.06) and recurrent falls (1.05, 1.03–1.07) after 5 years. Similarly, after 10-year follow-up (1.04, 1.01–1.07 and 1.07, 1.04–1.10; all *p* < 0.001). Increase in frailty at age 80, was similarly associated with an increased risk of falls after 5 years (1.04, 1.02–1.07, *p* < 0.001).

To understand how gradations of frailty associate with number of falls, we used frailty quintiles and four fall groups (Table 3). Already at baseline, the increment is stepwise between increasing frailty and number of falls, particularly pronounced for women having 2 and 3 or more falls within the 12 months prior to study inclusion.

**Table 3:** Gradients of frailty and number of women reporting none, one or multiple falls in different time perspectives. Frailty in quintiles at age 75 and falls^#^ prior to baseline, 5 and 10 years; frailty at age 80 and falls^#^ at 5 years

Frailty age 75 and incidence of women falling immediately prior to baseline*								
Frailty score at 75 y	No falls age 75		1 fall age 75		2 falls age 75		3 or more falls age 75	
≤ 0.10	167	(89.8)	14	(7.5)	5	(2.7)	0	(0.0)
0.11–0.14	145	(79.7)	19	(10.4)	12	(6.6)	6	(3.3)
0.15–0.19	134	(74.0)	27	(14.9)	12	(6.6)	8	(4.4)
0.20–0.27	117	(66.1)	30	(16.9)	13	(7.3)	17	(9.6)
0.28+	91	(48.4)	36	(19.1)	23	(12.2)	38	(20.2)

Frailty age 75 and incidence of women falling after 5 years**								
Frailty score at 75 y	No falls age 80		1 fall age 80		2 falls age 80		3 or more falls age 80	
≤ 0.10	111	(79.3)	11	(7.9)	10	(7.1)	8	(5.7)
0.11–0.12	105	(73.9)	17	(12.0)	11	(7.7)	9	(6.3)
0.13–0.17	95	(68.1)	28	(19.9)	12	(8.5)	5	(3.5)
0.18–0.24	96	(68.1)	18	(12.8)	14	(9.9)	13	(9.2)
0.25+	78	(55.7)	20	(14.3)	20	(14.3)	22	(15.7)

Frailty age 75 and incidence of women falling after 10 years**								
Frailty score at 75 y	No falls age 85		1 fall age 85		2 falls age 85		3 or more falls age 85	
≤ 0.09	50	(66.7)	13	(17.3)	9	(12.0)	3	(4.0)
0.10–0.11	45	(60.8)	15	(20.3)	8	(10.8)	6	(8.1)
0.12–0.15	41	(56.2)	17	(23.3)	10	(13.7)	5	(6.8)
0.16–0.21	31	(41.9)	17	(23.0)	11	(14.9)	15	(20.3)
0.22+	38	(50.7)	6	(8.0)	11	(14.7)	20	(26.7)

Frailty age 80 and incidence of women falling after 5 years***								
Frailty score at 80 y	NO FALLS age 85		1 fall age 85		2 falls age 85		3 or more falls age 8 y	
≤ 0.11	47	(68.1)	9	(13.0)	8	(11.6)	5	(7.2)
0.12–0.16	38	(54.3)	16	(22.9)	10	(14.3)	6	(8.6)
0.17–0.21	40	(58.0)	13	(18.8)	11	(15.9)	5	(7.2)
0.22–0.28	32	(45.7)	18	(25.7)	9	(12.9)	11	(15.7)
0.29+	30	(43.5)	10	(14.5)	10	(14.5)	19	(27.5)

^**#**^Falls occurring during the previous 12 months prior to each visit. Reported values, number(%). Chi-squared overall: *p < 0.001; **p = 0.001; ***p = 0.012

For the association between baseline frailty and future falls at age 80 and 85 the pattern is similar. With increasing frailty, the proportion of women falling increases, almost stepwise. In the highest frailty quintile, 15.7% had 3 or more falls at age 80, compared to 5.7% in the lowest quintile. After 10 years, more than one-quarter of women in the highest quintile sustained 3 or more falls (26.7% vs. 4.0%).

The association between frailty at age 80 and future falls at age 85 follows a similar pattern.

We combined and grouped women into fallers and non-fallers, investigating how frailty status affected their future-falls pattern. Among fallers at age 75, *regardless* of frailty status, approximately half had fallen at least once and one-third had recurrent falls at 5 years (Table 4). However, with reassessment at age 80, fallers who were *also frail*, fell more. Apart from a generally higher incidence at this age, frail women reported higher fall rates than non-frail, for at least one fall (76.9% vs. 57.3%) and recurrent falls (57.1% vs. 32.4%).

**Table 4:** Combined fall-frailty status and the relationship with frequency and odds risk of future falls in different time perspectives. Fall-frailty status at age 75 and falls^**#**^ at 5 and 10 years; fall-frailty status at age 80 and falls at 5 years

Combined falls-frailty and PROPORTION reporting falls^#^					
	At least 1 fall at 80 y			Recurrent falls at 80 y	
Fall-frailty status at 75 y	No(%) *n* = 631^a^		P^*^	No (%) *n* = 625^a^	P^*^
1. Faller *and* Frail	27 (54.0)	Group		18 (36.0)	
2. Faller *and* Non-Frail	53 (47.3)	1 v 2	0.432	39 (35.5)	0.947
3. Non-faller *and* Frail	22 (37.3)	3 v 4	0.035	16 (27.6)	0.0014
4. Non-faller *and* Non-Frail	100 (24.4)	1 v 4	< 0.0001	45 (11.1)	< 0.0001

	At least 1 fall at 85 y^#^			Recurrent falls at 85 y	
Fall-frailty status at 80 y	No (%) *n* = 358^a^		P^*^	No(%) *n* = 347^a^	P^*^
1. Faller *and* Frail	30 (76.9)	Group		20 (57.1)	
2. Faller *and* Non-Frail	43 (57.3)	1 v 2	0.039	23 (32.4)	0.015
3. Non-Faller *and* Frail	31 (48.4)	3 v 4	0.116	18 (28.1)	0.112
4. Non-Faller *and* Non-Frail	67 (37.2)	1 v 4	< 0.0001	33 (18.6)	< 0.0001

Combined falls-frailty and ODDS RISK of future falls				
	At least 1 fall^#^		Recurrent falls^#^	
	OR (CI 95%)	OR_adj_ (CI 95%)	OR (CI 95%)	OR_adj_ (CI 95%)
*FALLER and Frail at 75* *y*				
Risk of falling, 5 years (80 years)	1.31 (0.67–2.55)	1.14 (0.56–2.32)	1.02 (0.51–2.06)	0.83 (0.39–1.76)
Risk of falling, 10 years (85 years)	1.39 (0.51–3.82)	1.48 (0.49–0.46)	2.92 (1.08–7.91)	2.99 (1.03–8.67)
*NON-Faller and Frail at 75* *y*				
Risk of falling, 5 years (80 years)	1.84 (1.04–3.27)	1.95 (1.08–3.54)	3.06 (1.59–5.89)	3.24 (1.62–6.45)
Risk of falling, 10 years (85 years)	0.92 (0.37–2.30)	0.88 (0.33–2.35)	1.33 (0.46–3.86)	1.60 (0.53–4.82)
*FALLER and Frail at 80* *years*^b^				
Risk of falling, 5 years (85 years)	2.48 (1.03–5.95)	3.11 (1.10–8.78)	2.78 (1.21–6.41)	3.54 (1.37–9.12)
*NON-Faller and Frail at 80* *years*^b^				
Risk of falling, 5 years (85 years)	1.58 (0.89–2.82)	1.54 (0.83–2.87)	1.71 (0.88–3.31)	1.91 (0.94–3.87)

^**#**^Falls occurring during the previous 12 months prior to each visit

^a^Number of total cases with complete data

^b^Based on women age 80 and falls reported in the previous 12 months

*p values, Chi-squared. Odds ratios (OR) use non-frail category as reference. OR_adjusted_ for BMI, 25(OH)D, fractures, smoking

In a 10-year perspective (but not 5 years), women who were fallers *and* frail at age 75 were more likely to have recurrent falls at age 85 than their non-frail counterparts [2.92 (1.08–7.91)] (Table 4). *Fallers* at age 80 who were also frail had an increased risk for at least one fall [2.48 (1.03–5.95)] and for recurrent falls at age 85 [2.78 (1.21–6.41)].

Among non-fallers, frailty significantly impacts future falls. For non-fallers but frail at age 75, at least one fall and recurrent falls were both more frequent at age 80 (37.3% frail vs. 24.4% non-frail; 27.6% vs. 11.1%) (Table 4). The trend was similar, for women at age 80 and falls reported at 85. Estimating the risk, women who were non-fallers and frail at age 75 were three times more likely to have recurrent falls at age 80 [3.06 (1.59–5.89)] (Table 4). At age 80, the 5-year association between frailty and recurrent falls was, however, also non-significant [1.71 (0.88–3.31)].

The combination of being both frail and faller conferred a significantly higher risk of recurrent falls within 5 years compared to robust (non-faller, non-frail) women (age 75: 4.54, 2.35–8.71; age 80: 5.82, 2.79–12.56) in regression analysis using all four groups.

## Discussion

This study shows that in women, being frail at age 75 is a significant risk factor for recurrent falls both in five and 10-year perspectives. Frailty is a particularly strong predictor of future falls in women who have not yet experienced a fall, suggesting that if someone is frail, this is a time to intervene to avoid falls and fall-related injuries. In contrast, for women who have already experienced falls, frailty is secondary to prediction, most likely since they are already in the frailty–falls cycle.

The falls incidence increased between 75 y and 85 y (from one-third to almost half), with the most drastic change between 80 and 85, when the number of *individuals* falling increases, as does the number of *falls*. This precise change is difficult to capture in other studies [11, 22, 23]. Fall rates from 28.7 to 37.5% are observed in the National Health and Aging Trends Study, and while this is for somewhat younger ages including men, the 42.4% for age group 85–89 is consistent with our findings [24]. Age-related estimates of falls propensity are a foundation for understanding associated injuries; fractures being among the most important, although not part of this report.

The primary interest of this study is on recurrent falls as a sign of cumulative intrinsic age-related falls propensity. This is based on the assumption that frequent falling stems from failure of multiple physiologic systems, potentially captured by frailty, in contrast with the more arbitrary nature of one fall which may be accidental. To facilitate comparison with the existing literature, however, we also report ‘any fall’.

The reciprocity between frailty and falls is a major challenge to aging. With frailty increasing at each assessment age, those with the highest frailty had more falls in the previous year; and if highly frail, a higher incidence of future falls was also more likely.

Most studies find association between frailty and falls [1, 11, 15, 25, 26], although the relationship is unclear at the less-pronounced stages of frailty, reflecting the complexity in defining the transition from robust to pre-frail and frail. The strength of the association also varies depending on the age ranges, sex and setting of the studied populations [6]. An advantage in our setting is the single-age inclusion and duration of follow-up, which allows us to combine frailty and falls history to improve understanding of the interaction, albeit by 5-year increments. Hence, frailty has a long-term impact on falls, far beyond the one-to-3-year perspectives of existing studies, with women frail at age 75 having a continued higher falls propensity after 10 years compared to their non-frail counterparts.

A previous fall is a strong risk factor for future falls [3] which others have either adjusted for or performed subgroup analyses [25, 27]. To dissect the respective contribution from previous falls and frailty on the risk of future falls, we combined participants into fallers and non-fallers with or without frailty. Fallers and non-fallers have a distinctly different future-falls pattern. At age 75 frailty appears to be an important risk factor for women *without* a history of previous falls but not for women *with* falls. Conversely, at older ages frailty is a risk factor among *fallers* though not among *non*-*fallers*. We speculate that this is a consequence of the frailty–falls–frailty cycle and an indication of accumulated frailty with age. This is also obvious from the very different frailty score between frail and non-frail *non*-*fallers* at age 75 which is reflected in a higher recurrent falls risk after 5 years among the frail. Also, when reassessing frailty at age 80, the frailty score has a more normal distribution; the higher mean possibly reducing predictivity. One exisiting study of a mixed-sex population (mean 70.1 y) also reports that frailty is a stronger predictor in non-fallers, but another all-female survey (mean 69.4 y) reports the opposite [25, 28]; a likely explanation being that risk factors are both sex and age-specific.

As a way of understanding the transition to greater frailty and the association with falls, we examined initial frailty as a gradient, demonstrating a stepwise gradient in frailty quantifiable as an increasing number of falls at age 80 and 85. Although these women represent a relatively healthy susbset (having all survived 10 years and predominantly ‘non-frail’ or ‘pre-frail’), nevertheless differences in frailty are mirrored in the high proportion of recurrent fallers. This implies that a careful assessment of frailty in the elderly might be beneficial for fall prevention and possibly forestall the cycle of frailty and falls.

Strengths of this study include first that the participants are community-dwelling, older women of average health, at a pivotal phase, where detrimental changes accumulate at a higher rate. Therefore, this study also provides information essential for prevention strategies to reduce the impact and consequences of frailty. Second, since all women were identically aged at inclusion, confounding from chronological age is reduced and age-adjustment unnecessary. Third, the availability of data for 10 years and beyond allows us to assess fall risk with increasing frailty, providing a long-term perspective on the consequences of frailty for successful aging.

Limitations of the study are also acknowledged. First, for direct comparison to other studies use of the most widely used phenotypic definition of frailty by Fried et al. [1] would be preferable. However, since the cohort was designed to investigate bone health, not general health, in aging, this was impossible. Instead, following the rules of Searle et al. [20] we developed a frailty index which performs well [16, 17]. Second, one of the variables included in the index was ‘self-estimated fall risk’, since the index was constructed for use with multiple outcomes. However, this did not appreciably affect the results, without it associations were a little lower but still significant. Third, there is a risk for recall bias, since falls were self-reported. A 12-month period was decided to be an acceptable recall period, since the times between follow-up visits were long. It has, however, been suggested that a narrower time frame increases internal validity and that participants should be questioned about the past month [29] and the results should be interpreted with this in mind. In retrospect a design involving mailing post-cards or frequent telephone calls could potentially have decreased the risk of bias. Further to this, exact fall dates were not collected hence it is impossible to determine how many falls directly resulted in fracture or injury. Fall outcome was, however, beyond the scope of this study. Cognitive function and whether it affected fall recall was not specifically tested in the cohort. Fourth, longitudinal studies following older people have an inherent limitation of loss-to-follow-up, mainly because of morbidity, relocation or mortality. Among survivors, reasons for non-attendance in OPRA are described in detail elsewhere [19, 30] but briefly at 5-year follow-up this was primarily due to illness (31%), while other reasons included moving to a senior home, moving abroad, social reasons, mobility problems (16%). The remainder did not specify a reason. At 10-year follow-up illness accounted for 56% of those not attending, moving or other reasons (21%). We acknowledge that the length of follow-up and high age of the participants reduces the number of participants at each follow-up, an inherent problem in all stuch studies. However, since the incidence of falls increases with age the study is sufficiently powered.

In this population-based cohort of identically aged elderly women, frailty plays a significant role in the etiology of falls, most pronounced in those who are frail but have not yet reported a fall. It also emphasizes the connectivity between frailty and falls and the reciprocal increase in falls propensity and frailty status. These findings could be important in formulating prevention strategies, since it indicates that frailty assessment should be initiated early on.

## Electronic supplementary material

Below is the link to the electronic supplementary material.

Reports Odds Ratio (OR) calculated for frail women at age 75 and RECURRENT falls at 5 and 10 years; and frailty at age 80 and RECURRENT falls at 5 years (DOCX 45 kb)

Reports frailty across the duration of study and median F-index for those who FELL and those who did NOT at each visit (PDF 78 kb)
