# Supplementary material for: Frailty and prediction of recurrent falls over 10 years in a community cohort of 75-year-old women
Source: Aging Clin Exp Res. 2020 Jan 14;32(11):2241–50. doi: 10.1007/s40520-019-01467-1 (PMC7591409; doi:10.1007/s40520-019-01467-1)
Supplement: Supplementary file 1 — Reports Odds Ratio (OR) calculated for frail women at age 75 and RECURRENT falls at 5 and 10 years; and frailty at age 80 and RECURRENT falls at 5 years (DOCX 45 kb) [file 40520_2019_1467_MOESM1_ESM.docx]

# **Supplementary Table 1**. Frailty at age 75 and risk of RECURRENT falls at 5- and 10-years; and frailty at age 80 and RECURRENT falls at 5-years

|  | *No. with recurrent falls/No. in analysis* | OR (CI 95%) | *p* | OR_adj_ (CI 95%)* | *p* |
| --- | --- | --- | --- | --- | --- |
| **Frail at Age 75** |  |  |  |  |  |
| Risk of falling, 5-years (80y) | 124/704 | 2.55 (1.62-3.99) | <0.001 | 2.49 (1.55-3.99) | <0.001 |
| Risk of falling, 10-years (85y) | 98/371 | 3.04 (1.63-5.67) | <0.001 | 3.46 (1.78-6-73) | <0.001 |
|  |  |  |  |  |  |
| **Frail at age 80** |  |  |  |  |  |
| Risk of falling, 5-years (85y) | 94/347 | 2.14 (1.29-3.53) | 0.003 | 2.41 (1.39-4.15) | 0.002 |

*Odds Ratio (OR) calculated for frail women (Frailty Index ≥0.25); reference category is non-frail women (<0.25). *Model adjusted for BMI, 25(OH)D, fractures sustained between 50-75y and smoking habit.*
