# Supplementary figures and images for: Frailty and prediction of recurrent falls over 10 years in a community cohort of 75-year-old women
Source: Aging Clin Exp Res. 2020 Jan 14;32(11):2241–50. doi: 10.1007/s40520-019-01467-1 (PMC7591409; doi:10.1007/s40520-019-01467-1)

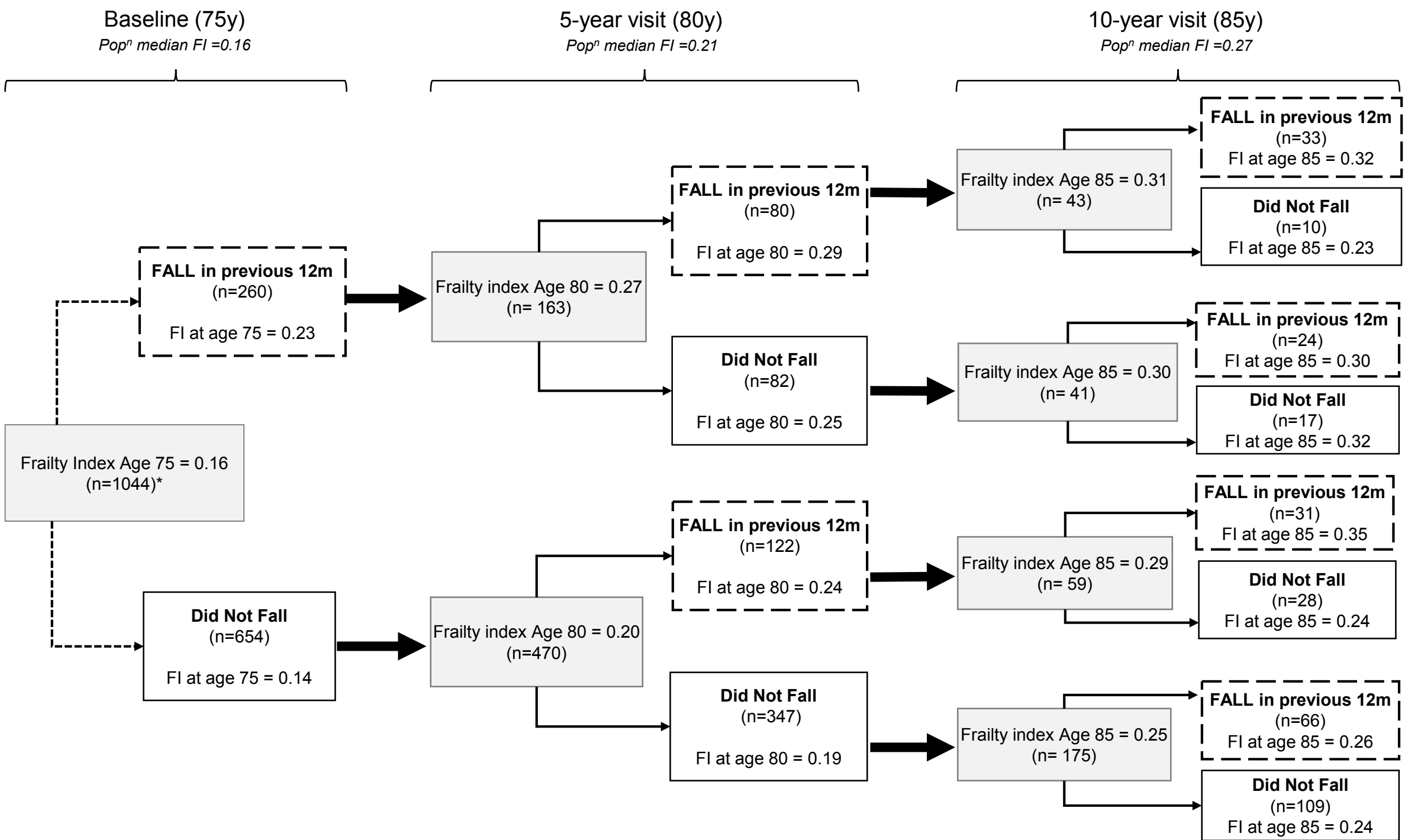

Supplement: Supplementary file 2 — Reports frailty across the duration of study and median F-index for those who FELL and those who did NOT at each visit (PDF 78 kb) [file 40520_2019_1467_MOESM2_ESM.pdf]
